# Editorial: Plant phenotyping for agriculture

**DOI:** 10.3389/fpls.2026.1904959

**Published:** 2026-06-19

**Authors:** Alejandro Isabel Luna Maldonado, Ajay Kumar, Yu Nishikawa, Wilgince Apollon

**Affiliations:** 1 Department of Agricultural and Food Engineering, Faculty of Agriculture, Autonomous University of Nuevo Leon, Nuevo Leon, Mexico; 2 Department of Biosystems and Agricultural Engineering, Oklahoma State University, Stillwater, OK, United States; 3 Faculty of Agriculture, Kagoshima University, Kagoshima, Japan

**Keywords:** computer vision, deep learning, high-throughput phenotyping, precision agriculture, unmanned aerial vehicle (UAV)

## 1 Introduction

Modern agriculture operates at an unprecedented crossroads, it must simultaneously accelerate crop yields to feed an expanding global population and adapt to the severe, fluctuating pressures of climate change, structural soil degradation, abiotic water deficits, and evolving biological threats. Historically, selecting resilient crop varieties and implementing field-scale management strategies relied extensively on destructive, labor-intensive, and fundamentally subjective visual metrics. This manual processing approach has long been recognized as the primary operational bottleneck in agricultural advancement.

To bridge the gap between rapidly expanding genomic data and actual field performance, the systematic, non-destructive quantification of structural and functional plant traits, plant phenotyping, has emerged as a transformative frontier. By integrating high-throughput engineering, multi-scale remote sensing, deep learning, and advanced molecular biology, modern phenotyping transitions crop science away from qualitative estimation toward highly reproducible, multi-dimensional data frameworks.

This Research Topic presents new advances in advanced 3D reconstruction and deep semantic segmentation at the seedling stage; amodal fruit segmentation, morphological extraction, and early water-stress diagnostics; high-throughput in-field seedling counting and dynamic density modeling; multimodal foundation models, network pruning, and intelligent phytoprotection; aerial and spaceborne remote sensing for canopy analysis and weed monitoring; plant physiology, functional spectroscopy, and functional genomics under abiotic stress; and automated diagnostics for real-time orchard scouting and vineyard management.

## 2 Advanced 3D reconstruction and deep semantic segmentation at the seedling stage

Automating the characterization of complex spatial layouts under controlled or greenhouse environments is essential for early variety selection and early-stage structural evaluation. Several contributions within this volume provide key breakthroughs in navigating overlapping tissues, severe occlusions, and low-contrast edge regions.

A prominent line of research leverages deep point-cloud architectures to preserve high-fidelity spatial geometry. Xu et al. address the persistent issue of spatial occlusion and complex field backgrounds in young plants by developing *EggplantPointNet++*. By pairing voxel-based background purification with DBSCAN clustering, their method provides high-precision 3D semantic segmentation and non-destructive phenotyping of eggplant seedlings, facilitating fully automated structural evaluations.

This methodology is further advanced and broadened by Yao et al., who propose a field phenotyping method for tobacco plants combining multi-view unmanned aerial vehicle (UAV) remote sensing with an enhanced *PointNet++* architecture. By integrating a Local Spatial Encoding (LSE) module and a Density-Aware Pooling (DAP) module, they achieved a mean Intersection over Union (mIoU) of 93.97% for tobacco stem and leaf segmentation, demonstrating that 3D point clouds can reliably compute key traits like plant height, leaf width, and internode length (R^2^ ranging from 0.86 to 0.95) under variable field topography.

Extending 3D structural analysis into greenhouse environments, Wang et al. introduce *VGDS-PointNet++*. By employing voxel grid downsampling with deep learning pipelines on tomato canopy point clouds acquired via depth cameras, their workflow automatically calculates leaf length and stem diameter, achieving an impressive mIoU of 88.95% and high correlation with manual validation datasets (R^2^ = 0.93).

Beyond early-stage seedlings, this architectural modeling extends to mature crop geometries in open fields. The development of *RAM-UNet* by Zhu et al. showcases how substituting standard convolutions with deformable convolutions enables deep neural networks to accurately isolate the main stem of mature, high-density crops like soybeans. This architecture overcomes the traditional challenges of color mimicry and severe occlusion by pods and leaves, achieving an outstanding mIoU of 90.58% and providing reliable indices for lodging resistance and structural yield modeling (R^2^ = 0.9746).

## 3 Amodal fruit segmentation, morphological extraction, and early water-stress diagnostics

Accurately extracting fruit morphology under commercial greenhouse conditions remains heavily constrained by overlapping crop structures, foliage cover, and variable shadows. Simple semantic masks typically fail when a target fruit is partially blocked, leading to a loss of key volumetric data.

To resolve the challenge of hidden boundaries, Li et al. developed CGA-ASNet, a specialized RGB-D amodal segmentation network driven by a Contextual and Global Attention (CGA) module designed to restore occluded tomato regions. Trained on a high-fidelity synthetic greenhouse dataset (*Tomato-sim*) generated via NVIDIA Isaac Sim’s Replicator Composer and optimized with a mean coordinate fusion algorithm for real-world validation, this architecture expands the network’s receptive field to predict the complete, hidden circular forms of occluded tomatoes, achieving an F@0.75 score of 94.2 and an amodal mIoU of 82.4%. This proves that simulation-to-real (Sim2Real) domain pathways can successfully decode full physical volumes under dense commercial canopies.

Complementing this structural restoration, Yang et al. designed an integrated diagnostic framework to identify early water stress dynamics in greenhouse tomatoes. Built upon an optimized *YOLOv11n* core, their system integrates adaptive kernel convolutions (AKConv) into the network backbone’s *C3k2* modules and implements a recalibration feature pyramid detection head based on the specialized P2 small-target layer. This combination achieved a 5.4% increase in mAP_50–_95 for identifying fine phenotypic parts. By applying automated geometric analysis to the extracted bounding boxes, the system extracts plant heights and petiole count with low relative errors, feeding these phenotypic parameters into a Random Forest classification routine that flags water-stressed plants with 98% accuracy to guide targeted, automated drip irrigation.

## 4 High-throughput in-field seedling counting and dynamic density modeling

Accurate plant stands during early vegetative stages represent the foundational metric required to establish true field emergence rates, validate seed vigor across diverse breeding blocks, and perform early yield predictions.

To solve the challenges of small targets, extreme spatial density, and adjacent leaf overlap, Zang et al. designed DM_IOC_fpn, a wheat seedling counting framework that balances local and global contextual features. By structuring a point-annotated dataset and embedding a density-enhanced encoder module, their network balances micro-scale spatial limits with macro-scale canopy structures. Optimized through a combined loss function tracking counting, classification, and regression parameters, this architecture achieved low error scores (RMSE = 2.91; MAE = 2.23), outperforming standard object-detection benchmarks in complex field environments.

At the same time, scaling up to real-time aerial monitoring required major reductions in model complexity to support resource-constrained edge computers on autonomous aerial platforms. Feng et al. engineered an ultra-lightweight YOLOv8n variant tailored for real-time maize seedling counting from high-speed UAV RGB overflights. By reparametrizing RepConv with HGNetV2, they constructed a lean *Rep_HGNetV2* backbone, integrated a Bidirectional Feature Pyramid Network (*BiFPN*) for multi-scale feature alignment, and implemented a Task Dynamically Aligned Detection Head (*TDADH*). This architecture compressed total model parameters by 47% and reduced weight sizes to 3.5 MB while maintaining a 96.5% detection accuracy and an ultra-fast processing speed of 146.3 FPS, paving the way for low-cost, real-time field scouting.

## 5 Multimodal foundations, network pruning, and intelligent phytoprotection

Automated phytoprotection requires machine-vision architectures capable of generalizing across highly diverse species, complex field conditions, and varying computational boundaries. A significant subset of the published papers addresses these challenges through foundation model adaptation, multi-modal alignment, and efficient network compression.

A major paradigm shift presented in this collection involves moving away from task-specific training and toward foundation model adaptation. Chen et al. introduce a novel architecture integrating the *DinoV3* foundation model with a Unet framework to achieve robust leaf lesion segmentation across diverse species (such as coffee and black gram). By incorporating a Spatial Prior Module (SPM), their approach surpassed standard benchmark networks by over 10.5% in IoU while reducing inference times by approximately 93.6%, demonstrating that high-parameter foundation models can be highly optimized for resource-constrained edge devices in real-time scouting.

To solve the perennial problem of limited training data for rare or emerging crop diseases, Cooper et al. developed an ingenious synthetic data generation pipeline. Combining 3D procedural leaf modeling in Blender with diffusion-based disease synthesis (*Stable Diffusion* fine-tuned with LoRA and ControlNet), they synthesized highly accurate plant disease images with perfect ground-truth annotation masks. When deployed in low-resource data settings, combining these synthetic pipelines with restricted real-world datasets consistently drives significant improvements in downstream segmentation tasks.

Furthermore, lightweight, high-throughput field classification models demonstrate the power of network pruning. Deployed on visible-light UAV platforms to screen extensive wild rice germplasm for Bacterial Blight (BB) resistance, Pan et al. developed *XooNet*. Utilizing the LAMP pruning strategy, they compressed model parameters to just 1.4M while retaining a 93.1% screening accuracy, drastically accelerating the high-throughput identification of valuable, wild genetic resources in field conditions.

In parallel, Zhao et al. presented a systematic review tracking the global trajectory of deep learning algorithms applied to foliar disease identification. Their work provides an explicit roadmap detailing how high-performance networks can mitigate real-world noise, such as erratic sunlight glare, multi-disease co-infection, and variable symptom morphology across overlapping infection stages.

To tackle specific, complex pathologies, Xu et al. developed the *TSSC* deep learning model, which embeds three-neighbor channel attention paired with a complementary squeeze-and-excitation mechanism. This specific architecture minimizes structural degradation risks while pushing classification accuracy to 99.61% for highly complex pea leaf pathologies. Similarly, Feng et al. tackled overlapping leaf occlusions and small lesion footprints in citrus groves with *YOLO-Citrus*, an optimized framework integrating C3K2-STA, ADown modules, and a Wise-Inner-MPDIoU loss function to strike a balance between edge computational constraints and field deployment.

## 6 Aerial and spaceborne remote sensing: canopy indices, weed dynamics, and generalization

UAVs and high-resolution satellite imagery have expanded the operational scale of phenotyping from individual pots to vast breeding blocks and commercial fields, allowing researchers to capture macro-dynamic parameters over time.

In complex canopy systems that defy standard top-down aerial sensing, such as single-staked white Guinea yams, Iseki et al. demonstrated the distinct advantage of utilizing multi-angle (combined nadir and oblique) UAV imaging configurations. When coupled with support vector regression, this method captures complementary canopy-structure information to model shoot biomass trajectories (R^2^ = 0.79) across multiple years and management zones. These non-destructive, time-series datasets enabled the fitting of genotype-specific Richard’s growth curves using Bayesian inference, isolating valuable genetic variations in early growth allocation.

To capture full-season vertical physiological changes over large scales, Li et al. developed a hybrid CNN-LSTM-Attention (CLA) model designed to estimate the full-period Leaf Area Index (LAI) in rice using multi-temporal UAV multispectral imagery. By using the CNN layer to extract instantaneous spatial features, the LSTM block to process seasonal time-series intervals, and a self-attention mechanism to weight critical growth transitions, their platform achieved a high coefficient of determination (R^2^ = 0.92) and kept relative root mean square errors (RRMSE) below 9%. This network minimized soil background noise during early vegetative stages (LAI values 1–3), outperforming standard machine learning benchmarks (SVR, PLSR, and XGBoost).

In precision weed management, tracking weed patches over time is critical for site-specific weed management (SSWM) to phase out uniform blanket herbicide spraying. Rosle et al. engineered a deep learning change-detection architecture using a Deep Feedforward Neural Network (DFNN) to perform automated post-classification comparisons of broadleaved weed infestations in rice fields over multiple drone flights. Their temporal maps tracked weed expansion from 40.95% at 34 days after sowing (DAS) to 47.43% at 48 DAS in untreated control plots. This precise spatial monitoring revealed a strong negative correlation (R^2^ = 0.9487) between weed coverage and herbicide-saving potential, confirming that early targeted drone scouting can save up to 40.95% in herbicide volume.

UAV multispectral analytics also proved highly effective at suppressing severe background interference (soil, drip-irrigation lines, and senescent leaves) during post-defoliant crop evaluations. In cotton breeding trials, Chen et al. proposed a UAV multispectral workflow for single-boll weight (SBW) estimation. By coupling object-based maximum likelihood classification for lint extraction with neural network regressions, their method effectively suppressed mixed-pixel background noise, achieving a validation coefficient of determination (R^2^) of 0.80 across diverse planting densities and cotton varieties.

Additionally, visible-light UAV semantic segmentation was applied to challenging environments like coastal or depleted margins. Wang et al. leveraged high-resolution visible imagery enriched with Self-Attention (SE) channel mechanisms to monitor Kenaf seedlings in saline-alkali soils. Achieving a high-precision canopy coverage segmentation (85.99% IoU), this automated method streamlined the field selection of stress-tolerant materials under commercial field conditions.

To resolve generalizability constraints when deploying historical field diagnostics to entirely new growing environments, Zhou et al. evaluated multi-source domain adaptation techniques using long-term UAV imagery of soybean breeding trials. Their work demonstrated that pre-training combined with target fine-tuning accurately isolates physiological maturity dates (R8 stage), achieving high agreement with ground-truth ratings (R^2^ = 0.74 to 0.79) and bounding root mean square errors below 2 days, proving that historical training datasets can be effectively transferred to unseen environments.

On a regional scale, the integration of spaceborne data was validated for macro-scale nutrient optimization. Morales-Ona et al. conducted field-scale evaluations of 3-m resolution PlanetScope satellite imagery to guide in-season precision nitrogen (N) management in corn across varying tillage and residue conditions. By examining the relationship between 16 distinct vegetation indices and true grain yield during critical vegetative windows (V10–V11), they established precise methods for estimating the Agronomic Optimum Nitrogen Rate (AONR_VI_), while highlighting how high crop residue can suppress spectral sensitivity, underscoring the absolute necessity of integrating localized agronomic context into satellite-driven diagnostic models.

## 7 Physiology, functional spectroscopy, and functional genomics under abiotic stress

Bridging the gap between an organism’s physical form, its physiological parameters, and its immediate micro-environment is a recurring theme across this Research Topic, emphasizing climate-resilient crop architecture and diagnostic spectroscopy under abiotic constraints.

Investigating heat stress mitigation in cereal breeding, Ruz-Ruiz et al. designed a high-throughput spectroradiometer phenotyping method to measure canopy albedo in wheat genotypes at the critical flowering stage. Their study revealed significant genotypic variations in canopy albedo under warm conditions, showing a direct relationship with canopy architecture and light interception (r = 0.74). While higher albedo was not linked to lower internal canopy temperatures, it significantly influenced the air temperature gradients across the vertical canopy profile, marking albedo as a valuable, additive structural trait for heat avoidance breeding in warming climate scenarios.

In precision weed management, advanced digital phenotyping platforms were thoroughly validated for pre-emergence herbicide dose-response assays. Landau et al. evaluated the non-destructive digital phenotyping system *Phenospex TraitFinder* to generate dose-response curves for common lambsquarters (*Chenopodium album L.*). Their work demonstrated that non-destructive Digital Biomass (DB) and 3D leaf area metrics generate growth-reduction curves (GR_50_) that are statistically equivalent to traditional, destructive fresh biomass measurements, allowing researchers to dramatically accelerate trial turnaround times while preserving valuable, herbicide-resistant biotypes.

Addressing specific nutrient management challenges in degraded landscapes, Khdery et al. integrated canopy hyperspectral reflectance with biochemical profiling to assess faba bean responses to varying foliar Zinc concentrations in sandy soils. Their results showed that optimal Zn enrichment (2.0g L^-1^) sharpens the red-edge slope (700–750 nm) and elevates near-infrared reflectance, offering a robust non-destructive diagnosis framework that links structural tissue responses directly to precision micronutrient interventions.

On a multi-modal level, Liang et al. pushed the boundaries of non-destructive stress screening by introducing Mm-VitnNet, a gated image-text interaction network designed to classify salt tolerance across 178 soybean varieties. By coupling chlorophyll fluorescence imaging phenotypes with textual parameter scripts through a learnable token mechanism, this network achieved an accuracy of 98.97%, demonstrating that cross-modal data alignment can mitigate inter-modality interference while conserving computational expenses (1.84 G FLOPs).

To tie these macroscopic phenotypic traits back to underlying genetic mechanisms, Chen et al. deployed long-term environmental phenotyping across a population of chromosome segment substitution lines (CSSLs) derived from *Glycine soja* and *Glycine max*. Across three unique environments, they mapped 130 quantitative trait loci (QTLs) governing vegetative growth intervals and reproductive yield metrics. This functional analysis isolated a critical target gene, GmFTIP09 within the *Chr09-cluster-1* locus. Knockout mutants of *Gmftip09* exhibited severe delays in both initial flowering (R1) and final crop maturity (R8), alongside substantial reductions in 100-seed weight (SW), confirming that this gene was a primary target of selection during modern crop domestication and providing a precise molecular target for marker-assisted breeding.

## 8 Automated diagnostics in real-time orchard scouting and vineyard management

Precision fruit tree cultivation requires robust identification mechanisms under dense canopy shade, erratic lighting conditions, and severe structural occlusions.

To address scientific thinning requirements in orchards, Ji et al. developed the MDI-YOLOv11 architecture for the real-time detection and density estimation of peach tree inflorescences. By incorporating an RFCAConv module into the network backbone and embedding a P2 layer with a RepGFPN structure, their platform successfully navigated high-density flower and bud occlusions, securing an AP_50_ of 0.919 with a rapid processing inference time of 13.46 ms per frame, and automatically generated row-by-row density distribution maps to guide targeted orchard interventions.

To optimize precision table grape vineyard management, Du et al. introduced a dual-branch network named *MVDNet* alongside a post-processing algorithm for in-cluster grape berry counting and cluster segmentation. Their model achieved a high coefficient of determination (R^2^ = 0.886) while utilizing only 3.372 million parameters, making it uniquely suited for edge deployment on low-power devices to automate automated berry thinning.

Maintaining and tracking the developmental stages throughout the entire life cycle of plants is also a core focus of intelligent greenhouse research. Kim et al. designed a physiologically grounded pipeline for multi-plant basil environments that monitors the emerging number of leaf pairs at the shoot apex. By pairing top-view camera arrays with YOLO object detection and K-means spatial alignment, their regression model achieved a high performance (R^2^ = 0.96; MAE = 0.13), enabling automated growth-stage classification above 98% without relying on rigid, error-prone time-based criteria.

## 9 High-throughput grain metrics, variety identification, and postharvest automation

Completing the continuum from cellular morphogenesis to industrial crop processing and postharvest storage, multiple works focus on final yield architecture, reproductive trait tracking, and automated sorting networks.

In cereal crop assessment, Wang et al. established a high-throughput deep learning framework targeted at reproductive structures, introducing the OPG-YOLOv8 model for multi-trait extraction in rice panicles. Paired with a dynamic depth-first search (DFS) pruning algorithm, the workflow successfully extracted panicle lengths (R^2^ = 0.9583) and structural grain dimensions across distinct maturity stages, executing at an inference speed of 154 FPS. Combined with Hunter Lab color calculations for continuous maturity indexing, this end-to-end framework bridges field visual tracking with direct yield determinants.

On a similar note, tracking early lifecycle development remains paramount for staple crops. Hou et al. presented SeedRuler, a web-based platform tailored for high-efficiency rice seed germination analysis. By integrating a traditional image processing module with deep-learning-based object detection (*SeedRuler-YOLO*) and fine-grained segmentation via the Segment Anything Model (*SeedRuler-SAM*), this platform successfully automates seed size measurements and customized germination tracking in under 30 seconds per image (mAP = 0.955).

Field grain quantification also saw substantial field-level upgrades through the work of Li et al., who developed YOLOv11-EDS for real-time field counting of wheat ears. By incorporating the DySample dynamic upsampling operator, an Efficient Channel Attention (ECA) mechanism, and a Slim-Neck feature fusion structure, their model reduced total parameters to 2.5M while expanding mAP_50_ by 1.5 percentage points on global validation datasets.

Beyond monocots, variety identification and DUS (Distinctness, Uniformity, and Stability) validation methods were reinforced by Zhang et al. They established a multidimensional framework for eggplant fruits, combining CIELAB color profiles with DNA fingerprinting and K-means clustering to explicitly isolate color shifts during physiological ripening. Similarly, Yang et al. tackled high-precision sorghum seed variety identification by constructing an integrated image-hyperspectral fusion dataset tracking 12,800 seeds across 8 distinct varieties. By embedding Deep Separable Convolutions (DSC) and a Convolutional Block Attention Module (CBAM) into a ResNet architecture, their *CBAM-ResNet50-DSC* model classified identical-looking seed lines with 94.84% accuracy, providing a non-destructive method for commercial seed quality assurance.

In the realm of high-value industrial crops, deep learning was applied to automated processing lines and indoor spawn propagation. Luo et al. introduced YOLO-AE, an improved model incorporating ACmix and EUCB layers for white tea precision winnowing equipment. This model cut processing inference times by 40% while tracking grade parameters in real time to automatically guide mechanical wind selection benches.

For mushroom breeding, Zhao et al. built an integrated machine vision pipeline utilizing an ACmix-ADown-YOLOv11n core coupled with EfficientSAM to compute 12 key structural traits of shiitake stipes, tracking centerline coordinates with extreme precision (R^2^ = 0.992). This specific work was complemented by Li et al. (2025)., who introduced VG-Stick-YOLOv11 paired with a ResNet-Stick-UNet (RS-UNet) configuration to map and calculate browning area ratios on shiitake cultivation sticks, providing a quantitative metric for substrate maturity.

Finally, bridging structural analytics with physiological postharvest degradation, Wang et al. conducted an exhaustive biochemical evaluation of postharvest apple flesh browning under low-temperature storage. Their research verified that prolonged cold stress triggers ROS-mediated (H_2_O_2_ and O_2_) membrane compartmentalization collapse, driving a 2.6-fold increase in polyphenol oxidase (PPO) activity. This metabolic breakdown loop establishes an empirical biochemistry foundation that directly mirrors the macroscopic color changes tracked by the visual non-destructive sorting models described throughout this volume.

To catalyze global adoption and lower the entry barrier for these multi-sensor methods, Parker et al. delivered a comprehensive open-access methodology repository and educational framework. Through their structured web platform (*PlantScienceDroneMethods.github.io*) and a dedicated technical training channel, they provided the plant science community with complete, end-to-end protocols, example flight data, and open-source scripts designed to convert raw multi-sensor aerial data into clean, tabular phenotypic matrices. This effort removes major computational bottlenecks and ensures that high-throughput phenomics remains accessible to researchers worldwide.

## 10 Conclusion

The future of agricultural sustainability depends on the maturity, integration, and scaling of advanced plant phenotyping. By moving past the constraints of manual, subjective data collection, unified workflows, ranging from 3D point-cloud processing and foundation vision models to multi-view drone telemetry and high-resolution satellite agronomy, provide the tools required to dissect complex genotype-by-environment (G × E) interactions. As these advanced diagnostic tools continue to mature, reduce computational demands, and integrate with open-access frameworks, they will accelerate the selection of climate-resilient cultivars, optimize resource-use efficiency, and safeguard global food security through data-driven, intelligent decisions.

In addition, modern plant phenotyping has become a strategic pillar for smart agriculture. The integration of advanced sensors, computer vision, deep learning, multimodal models, and remote sensing platforms transforms plant observation into a quantitative, scalable, and highly reproducible process. These capabilities enhance decision-making for water and nutrient management, disease control, yield optimization, and automation of production systems. Widespread adoption of these tools, supported by open-access frameworks and efficient computational solutions, will be key to fostering resilient crops, sustainable practices, and global food security under changing climatic conditions.

